# Shared genetic etiology underlying Alzheimer’s disease and major depressive disorder

**DOI:** 10.1038/s41398-020-0769-y

**Published:** 2020-03-09

**Authors:** Michael W. Lutz, Daniel Sprague, Julio Barrera, Ornit Chiba-Falek

**Affiliations:** 1 Division of Translational Brain Sciences, Department of Neurology, Duke University Medical Center, Durham, NC USA; 2 Center for Genomic and Computational Biology, Duke University Medical Center, Durham, NC USA

**Keywords:** Molecular neuroscience, Depression, Comparative genomics, Clinical genetics

## Abstract

Patients with late-onset Alzheimer’s disease (LOAD) frequently manifest comorbid neuropsychiatric symptoms with depression and anxiety being most frequent, and individuals with major depressive disorder (MDD) have an increased prevalence of LOAD. This suggests shared etiologies and intersecting pathways between LOAD and MDD. We performed pleiotropy analyses using LOAD and MDD GWAS data sets from the International Genomics of Alzheimer’s Project (IGAP) and the Psychiatric Genomics Consortium (PGC), respectively. We found a moderate enrichment for SNPs associated with LOAD across increasingly stringent levels of significance with the MDD GWAS association (LOAD|MDD), of maximum four and eightfolds, including and excluding the *APOE*-region, respectively. Association analysis excluding the *APOE*-region identified numerous SNPs corresponding to 40 genes, 9 of which are known LOAD-risk loci primarily in chromosome 11 regions that contain the *SPI1* gene and *MS4A* genes cluster, and others were novel pleiotropic risk-loci for LOAD conditional with MDD. The most significant associated SNPs on chromosome 11 overlapped with eQTLs found in whole-blood and monocytes, suggesting functional roles in gene regulation. The reverse conditional association analysis (MDD|LOAD) showed a moderate level, ~sevenfold, of polygenic overlap, however, no SNP showed significant association. Pathway analyses replicated previously reported LOAD biological pathways related to immune response and regulation of endocytosis. In conclusion, we provide insights into the overlapping genetic signatures underpinning the common phenotypic manifestations and inter-relationship between LOAD and MDD. This knowledge is crucial to the development of actionable targets for novel therapies to treat depression preceding dementia, in an effort to delay or ultimately prevent the onset of LOAD.

## Introduction

Patients with late-onset Alzheimer’s disease (LOAD) frequently manifest comorbid neuropsychiatric symptoms (NPS), with depression and anxiety being most prevalent^1–4^. Furthermore, depression has been found to be associated with increased risk to develop LOAD^5–9^. Major depressive disorder (MDD) is a neuropsychiatric condition, and patients with MDD, especially in late life, exhibit cognitive deficits and have an increased incidence of LOAD^10^. Thus, MDD may be a risk factor for LOAD, and/or part of the heterogeneity of NPS in LOAD^11,12^. These lines of evidence suggest the possibility of shared etiologies and intersecting pathways between LOAD and MDD.

The pathogenesis of both LOAD and MDD is complex and involves polygenic risk factors. Several studies suggested that risk genes for MDD may be involved in LOAD^13–15^. Genome-wide association studies (GWAS) identified numerous loci associated with the risk to develop LOAD^16–22^ and MDD^23,24^. These large publicly accessible GWAS data sets could be leveraged to facilitate investigations of whether the comorbidity and risk inter-relationship of these disorders can be explained by common genetic variants. Recently, a statistical method to evaluate genetic pleiotropic effects using GWAS summary statistics (*P-*values and odds ratios) was developed^25–28^, and has been utilized to examine genetic pleiotropy between multiple diverse diseases and phenotypes^25–27,29–35^, including LOAD with other conditions. Most prominent, polygenic overlaps were reported for LOAD with modulation of c-reactive protein (CRP) and plasma lipids^28,36^, and with type 2 diabetes^37^. Here, we utilize this statistical framework to investigate the genetic overlap between LOAD and MDD.

To date, the underpinning genetics of NPS heterogeneity in LOAD and of depression and LOAD comorbidity are yet to be discovered. While it has been shown that MDD-associated genes may contribute to LOAD^13–15^, a recent study found no evidence to support a common polygenic structure for LOAD and MDD^38^. Nonetheless, the genetic pleiotropy between LOAD and neuropsychiatric disorders, including MDD, has been understudied. In this study, we characterized the genetic signatures that are shared between LOAD and MDD. We undertook a robust new statistical strategy that integrates results from large multicenter meta analyses of LOAD and MDD GWAS to identify genetic variants and genes that are associated with LOAD conditional on an association with MDD. Our analysis pipeline progresses from identification of pleiotropic single-nucleotide polymorphisms (SNPs) common to the two conditions, through the genomic loci tagged by the SNPs and the candidate genes within the associated region, and ultimately, biological pathways.

## Materials and methods

### GWAS data sets

GWAS summary statistics were obtained from publicly accessible web sites for the LOAD GWAS and the MDD GWAS (see “Data availability”).

The LOAD GWAS data set consisted of summary statistics of *P*-values, beta coefficients, and standard errors, effect alleles from the International Genomics of Alzheimer’s Disease Project (IGAP)^22^. IGAP is a large three-stage study based upon GWAS on individuals of European ancestry. Stage 1 IGAP results were used, which included genotyped and imputed data on 11,480,632 single-nucleotide polymorphisms (SNPs) from 21,982 Alzheimer’s disease cases and 41,944 cognitively normal controls from four consortia: The Alzheimer Disease Genetics Consortium (ADGC); The European Alzheimer’s disease Initiative (ELOADI); The Cohorts for Heart and Aging Research in Genomic Epidemiology Consortium (CHARGE); and The Genetic and Environmental Risk in LOAD Consortium Genetic and Environmental Risk in LOAD/Defining Genetic, Polygenic and Environmental Risk for Alzheimer’s Disease Consortium (GERAD/PERADES).

The MDD GWAS data set (PGC-MDD2) consisted of summary statistics of *P*-values, odds ratios and standard errors, reference allele, imputation quality score (INFO), and direction of effect in each cohort from the Psychiatric Genomics Consortium^39^. The results were obtained for five cohorts described by Wray et al.^39^ (deCODE, Generation Scotland, GERA, iPSYCH, and UK Biobank), excluding the Hyde et al. cohort^24^ (23and Me, Inc.). These results included genotyped and imputed data on 13,554,550 variants from 59,851 MDD cases and 113,154 controls. Restricting variants to SNPs with high-quality imputation scores (0.6 ≤ INFO < 1.06) resulted in a total of 9,154,389 SNPs in common for the two data sets that were used for further analysis. Details for the genotyping procedure, quality control, and GWAS analysis are provided in the primary papers for the LOAD GWAS^22^ and the MDD GWAS^39^. All genomic coordinates are based on NCBI Build 37/UCSC hg19.

### Statistical and bioinformatics analysis

#### Pleiotropy analysis

The pleiotropy analysis strategy, based on conditional false discovery rates, fold-enrichment plots, and conditional quantile–quantile (Q–Q) plots, is described in detail elsewhere^28,37^. In brief, for two phenotypes A and B, pleiotropic enrichment of phenotype A conditional on phenotype B exists if the proportion of variants (SNPs) statistically significantly associated with phenotype A increases as a function of increased statistically significant SNP associations with phenotype B. The number of SNPs associated with phenotype A was determined for several thresholds of SNP association with phenotype B; the proportions were calculated relative to a baseline of all SNPs statistically significantly associated with phenotype A. For this study the analysis was run in both directions, with primary phenotype A as late-onset Alzheimer’s disease, and conditional phenotype B as MDD, followed by interchange of the primary and conditional phenotypes. Fold-enrichment plots graphically depict pleiotropy by showing fold enrichment in terms of numbers of SNPs on the ordinate and nominal –log_10_(*P*) values for association with cognitive impairment on the abscissa. Separate curves were shown for subsets of SNPs that reach specific levels of significance for their association with MDD, respectively. Conditional quantile–quantile plots for the same data shown in the fold-enrichment plots provided additional assessment of genetic pleiotropy for each set of GWAS results. Following the prior analysis strategy^28^, we focused the analysis for polygenic enrichment on SNPs below the standard GWAS Bonferroni-corrected *P*-value thresholds. Following the example of Wang et al.^37^ and Desikan et al.^28^, the SNP data were pruned to eliminate correlated pairs of SNPs based on linkage disequilibrium (LD) measured in the 1000 Genomes data set (Phase 3, version 5 of the 1000 Genomes Project, European panel). If *R*^2^ value for a pair of SNPs was >0.2, the SNP with the lower minor allele frequency (MAF) was removed.

For identification of specific SNPs conditionally associated with LOAD and MDD, a conditional false discovery rate (FDR) statistic (*Q* value) was calculated as described in the prior implementation of this analysis strategy^28,37^ and other publications^25–27,30–33^. This framework was an extension of the standard analysis for FDR calculations and uses information from the secondary phenotype to re-rank the *P*-values for the primary phenotype. The value of the conditional FDR for each SNP was calculated in the case where LOAD is the principal phenotype conditioned on MDD (LOAD|MDD) as well as the reverse (MDD|LOAD). We used a conditional FDR of *Q* < 0.05 to show statistical significance. The significance threshold of *Q* = 0.05 for the conditional FDR^40^ corresponds to 5 false positives per 100 reported associations. Manhattan plots of the conditional FDRs for were used to summarize the data.

In order to detect common susceptibility loci for LOAD and MDD after calculating the conditional FDRs (*Q* values) for each SNP under LOAD|MDD and MDD|LOAD, we computed the conjunction conditional FDR, which refers to the probability that a given SNP is null for both phenotypes. The conjunction conditional FDR (ccFDR) is the maximum value of the two conditional FDR (*Q*) values. A Manhattan plot was produced for the conjunction conditional FDR.

#### Mendelian randomization analysis

To test for a causal relationship between the set of SNPs identified by the conditional analysis, we performed Mendelian randomization (MR) analysis^41^ using the LOAD GWAS SNPs as instrumental variables, LOAD as the exposure, and MDD as the outcome. The LOAD GWAS SNPs were used to define the instrumental variables. These SNPs are highly replicated, share rigorous genetic associations with LOAD, and are randomly distributed in the general population with respect to lifestyle and environmental factors. Analysis of horizontal pleiotropy, where the SNPs associate with LOAD but influence MDD through pathways that are not specific to AD (e.g. the exposure) was completed. Two sample MRs^42^ were used for the statistical analysis using the MR-Base resource^43,44^. The MR Egger methodology^45^ was used for all calculations. For the exposure (LOAD), default parameter settings of a *P*-value threshold of 1 × 10^−8^, LD *R*^2^ of 0.001, clumping distance of 10 kb were used.

#### Functional genomics bioinformatics analyses

Functional bioinformatics analysis was performed to evaluate the biological significance of the SNPs that were identified in the pleiotropy analysis as showing conditional association between LOAD and MMD. Two bioinformatics analysis tools were used to map the SNPs to genes by proximity, define the genomic context for the variant, annotate effects on phenotypes, and identify relevant literature about the variant. The UCSC genome browser (http://genome.ucsc.edu/) was used to map each variant to proximate genes and to provide the first level of information about the genes and biological consequences of the genes^46^. SNPnexus (http://www.snp-nexus.org/) was used to provide additional annotation on gene/protein consequences and phenotype- and disease-association for the variants^47,48^.

Gene set enrichment and pathway analysis was completed using *i*-Gsea4Gwas^49^. This analysis was run on all SNP association results for the IGAP discovery data set and the MDD replication data set using the FDR *Q* values *Q*(LOAD|PSTD). The SNP to gene mapping was limited to 500 kb upstream and downstream of the gene. Candidate gene sets included canonical pathways, GO biological processes, and GO molecular function.

Gene expression analysis and clustering was performed using the GENE2FUNC capability of the FUMA GWAS analysis suite^50^. Gene expression data for the 53 tissue types was from Genotype-Tissue Expression (GTEx) v6, for the whole blood from the GTEx portal v7 data release^51,52^, and for the monocytes from the Cardiogenics study^53^.

eQTL analyses were performed on the GTEx portal using whole-blood data from GTEx portal v7 data release, and using a SAS macro for the monocyte data obtained from the Cardiogenics study^53^. For the latter, we applied a Bonferroni level of 5.4 × 10^−7^ for significance.

Proxy SNPs (*D*’ = 1, *R*^2^ > 0.9) were found using the NIH LDproxy tool^54^ in the CEU and GBR populations (https://ldlink.nci.nih.gov/?tab=ldproxy).

## Results

### Genome-wide association summary results for LOAD and MDD

Prior to assessment of polygenic overlap between LOAD and MDD, the individual GWAS for each phenotype was compared for quality control (QC) and overall genetic association statistics. QC details are reported in the primary publications for IGAP LOAD^22^ and for the Psychiatric Genomics Consortium MDD GWAS (PGC-MDD2)^39^. Genomic inflation was well controlled in both of these GWAS, and the minor allele frequencies (MAF) were limited to MAF > 0.01. Genome-wide significance levels were set at *P* ≤ 5 × 10^−8^ for both the LOAD GWAS and for the MDD GWAS based on Bonferroni corrections for the number of SNPs.

Inspection of the Manhattan plots (Supplementary Fig. S1) show several regions of the genome with nominal levels of association (*P* ≤ 1 × 10^−5^) for the different phenotypes, LOAD and MDD. The *Q*–*Q* plots (Supplementary Fig. S2) show that population stratification was accounted for in the association analysis.

### Assessment of polygenic overlap between LOAD and MDD

#### Genome-wide fold enrichment

**Fig. 1:**
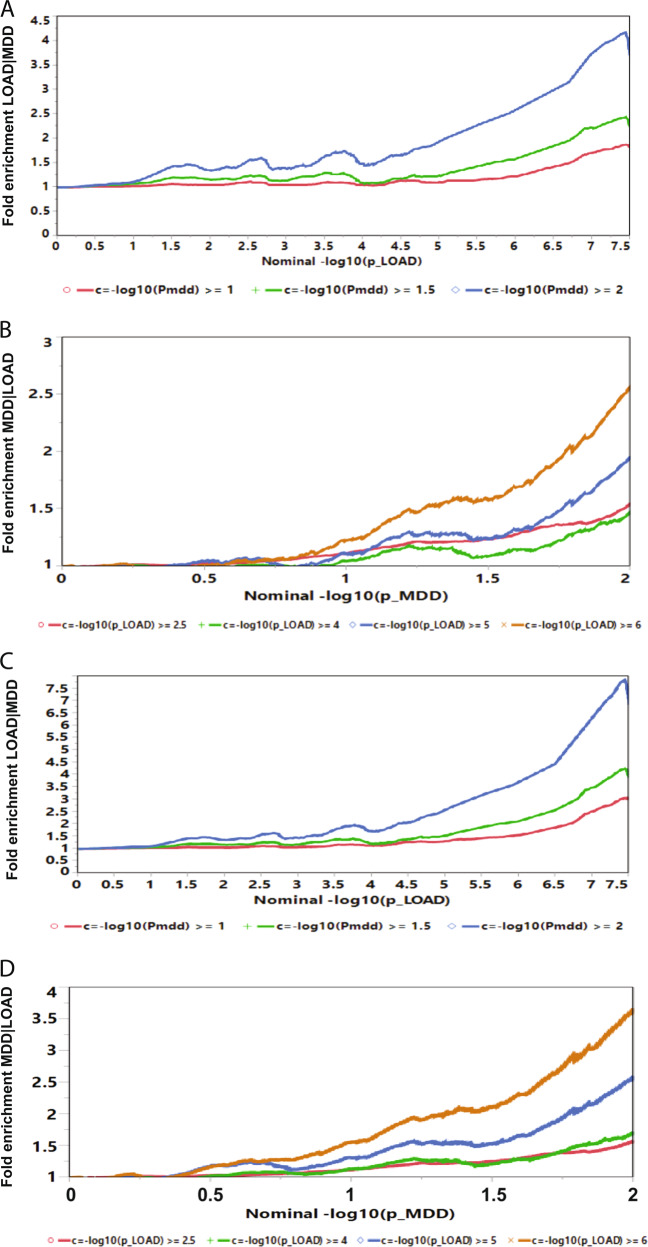
Fold-enrichment plots. Ordinate is fold enrichment. **a** Abscissa is nominal –log_10_(*P*) for SNP association with LOAD, curves are differentiated by the threshold for level of statistical significance for SNP association with the secondary phenotype (MDD). Plot is made for the results from the full genome. **b** Abscissa is nominal –log_10_(*P*) for SNP association with MDD, curves are differentiated by the threshold for level of statistical significance for SNP association with the secondary phenotype (LOAD). Plot is made for the results from the full genome. **c** Abscissa is nominal –log_10_(*P*) for SNP association with LOAD, curves are differentiated by the threshold for level of statistical significance for SNP association with the secondary phenotype (MDD). Plot is made for the results excluding SNPs in the *APOE*-associated region. **d** Abscissa is nominal –log_10_(*P*) for SNP association with MDD, curves are differentiated by the threshold for level of statistical significance for SNP association with the secondary phenotype (LOAD). Plot is made for the results, excluding SNPs in the *APOE*-associated region.

The fold-enrichment plot demonstrated SNP moderate (2.0–4.2-fold) enrichment for LOAD across increasingly stringent levels of significance with the MDD GWAS association (LOAD|MDD) (Fig. 1a). The reverse conditional association (MDD|LOAD) showed enrichment of ~1.8–7.0-fold (Fig. 1b). These results support a moderate level of polygenic overlap between LOAD and MDD.

#### Fold enrichment excluding the *APOE* region on chromosome 19

The chromosome 19 region containing the *APOE* gene is well-established as a strong genetic risk factor for LOAD, with GWAS association *P*-values on the order of 10^−238^ to 10^−12^ in the IGAP data set. Since the objective of the study was to find pleiotropic variants for LOAD and MDD, and the MDD data sets did not have any variants close to the magnitude of the *APOE* level of association (strongest association was *P* = 2.3 × 10^−11^), a second set of analyses excluding variants in the *APOE* region were performed. Variants in the *APOE* region on chromosome 19, defined as ± 300 Kb of the *APOE* epsilon coding SNPs (chr19:45,111,942–45,711,941) were excluded. The fold-enrichment plot demonstrated moderate (2.4–7.8-fold) SNP enrichment for LOAD across increasingly stringent levels of significance with the MDD GWAS association (LOAD|MDD) (Fig. 1c). The reverse conditional association (MDD|LOAD) showed enrichment of ~2.0–7.2-fold (Fig. 1d). These results support a moderate level of polygenic overlap between LOAD and MDD. Excluding the SNPs in the *APOE* region clearly identified a stronger polygenic overlap in the direction LOAD|MDD, with maximal fold enrichment increasing from approximately fourfold (including *APOE*) to eightfold (excluding *APOE*), while maximal fold enrichment in the reverse direction, (MDD|LOAD) was similar at sevenfold for analyses that included or excluded the SNPs in the *APOE* region. These results support the conclusion that including the *APOE* region for the conditional analyses can potentially mask significant results by establishing a set of *APOE* SNPs with a significant FDR that offsets other SNPs with FDRs that do not reach the level of the *APOE* SNPs.

#### Mendelian randomization analysis

The MR analysis estimated a moderate influence of the LOAD-associated SNPs on risk of MDD: OR = 1.4, 95% confidence limits 1.2–1.7. These results support that the genetic risks associated with Alzheimer’s disease may influence the risk of MDD. To test the sensitivity of the results to horizontal pleiotropy, we performed MR regression. The test for directional horizontal intercept was not significant (*P* = 0.09).

### Specific variants and genes identified by conditional false discovery rate analysis

**Fig. 2:**
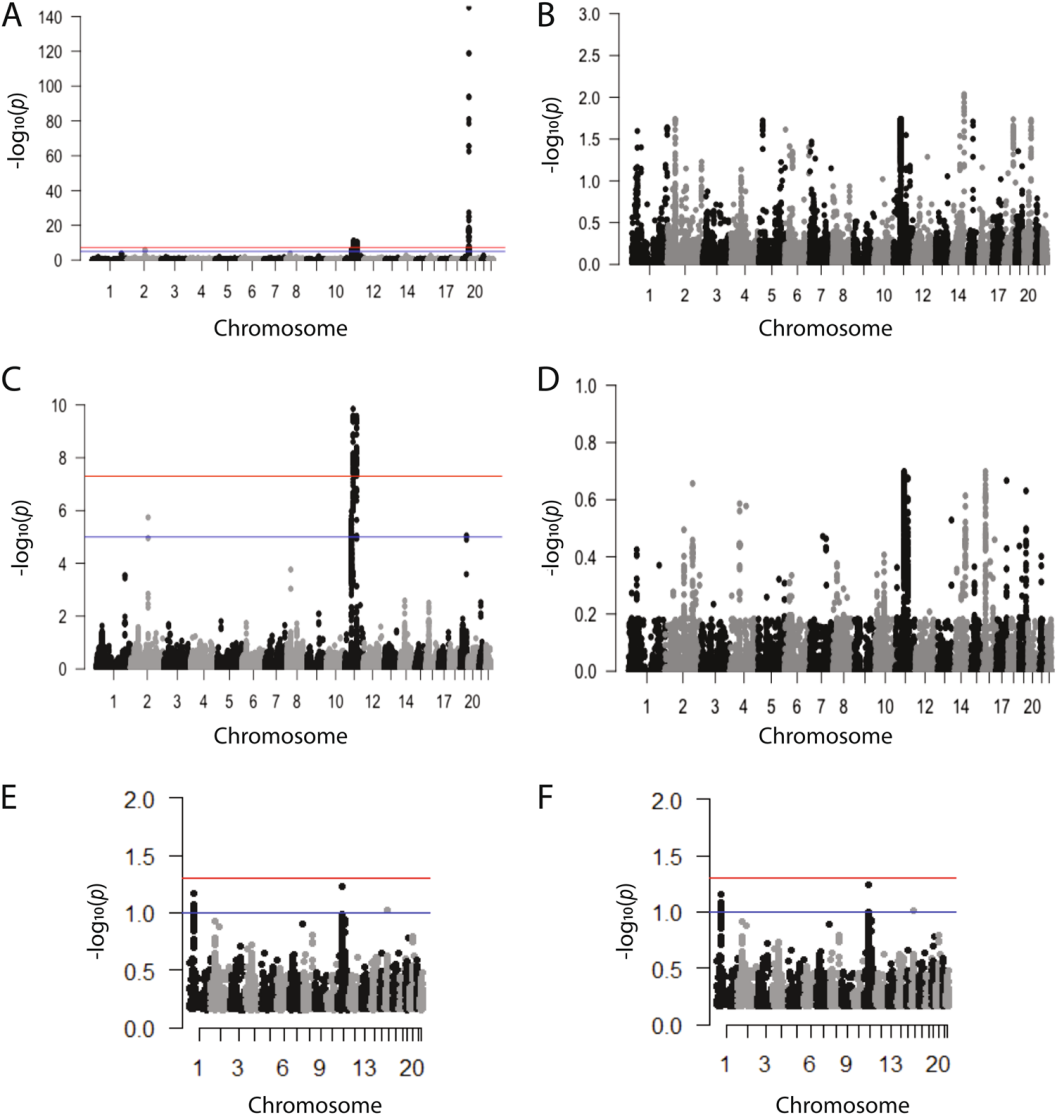
Conditional Manhattan plots of the conditional –log_10_ (FDR) values. **a** FDR *Q* value for SNP association with LOAD conditional with SNP association for MDD. Plot is made for the results from the full genome. **b** FDR *Q* value for SNP association with MDD association conditional with SNP association for LOAD. Plot is made for results from the full genome. **c** FDR *Q* value for SNP association with LOAD association conditional with SNP association for MDD. Plot is made for the results, excluding SNPs in the *APOE*-associated region. **d** FDR *Q* value for SNP association with MDD association conditional with SNP association for LOAD. Plot is made for the results, excluding SNPs in the *APOE*-associated region. **e** conjunction conditional FDR *Q* value for SNP association with LOAD conditional with SNP association for MDD. Plot is made for the results, including SNPs in the *APOE*-associated region. **f** Conjunction conditional FDR *Q* value for SNP association with MDD conditional with SNP association for LOAD. Plot is made for the results, excluding the *APOE*-associated region. For panels **a**–**d**, genome-wide significant line (red) is drawn at –log_10_(5 × 10^−8^), suggestive line (blue) is drawn at −log_10_(1 × 10^−5^); for panels **e** and **f**, genome-wide significant line (red) is drawn at –log_10_(0.05), suggestive line (blue) is drawn at –log_10_(0.1).

Manhattan plots based on conditional FDR analysis were constructed for data sets including (Fig. 2a, b) and excluding (Fig. 2c, d) the *APOE* region. The conditional association analysis excluding the *APOE* region, in which LOAD is conditional on association with MDD, *Q*(LOAD|MDD), identified a highly significant (FDR *Q* ≤ 5 × 10^−8^) cluster of SNPs on chromosome 11. In addition, two SNPs on chromosome 2 and two SNPs on chromosome 19 were identified as significant at a threshold of FDR *Q* ≤ 1 × 10^−5^ (Fig. 2c, Table 1). Relaxing the FDR threshold to *Q* ≤ 0.05 (−log_10_(*Q*) = 1.3) resulted in numerous SNPs across the genome (Table 1). The associated SNPs were classified as common characterized by 0.02 ≤ MAF ≤ 0.90. Individual GWAS results for these SNPs were consistent with effect sizes for GWAS studies of complex diseases: LOAD (IGAP): 0.83 ≤ OR ≤ 1.12, 8.67 × 10^−5^ ≤ *P* ≤ 3.15 × 10^−3^; MDD: 0.86 ≤ OR ≤ 1.07, 6.31 × 10^−7^ ≤ *P* ≤ 6.14 × 10^−4^. Mapping the most proximate genes to the associated SNPs defined 40 genes of which 9 have been known as LOAD risk genes via published LOAD-GWAS: *BIN1*, *CELF1*, *CR1*, *FERMT2*, *MS4A6A*, *PICALM*, *PTK2B*, *SORL1*, and *SPI1*.

**Table 1:** Genes reaching statistical significance (FDR ≤ 0.05) for association with LOAD conditional on association with MDD.

SNP	Chr.	Position^a^	Nearest gene	minor allele	MAF	Conditional FDR Q(LOAD|MDD)	LOAD Beta	LOAD Beta SE	LOAD *P*-value	MDD OR	MDD OR SE	MDD *P*-value
rs35677603	11	47,268,310	ACP2	T	0.31	1.58E−03	−0.078	0.016	4.05E−07	0.985	0.009	8.59E−02
rs3017432	21	28,147,728	ADAMTS1	T	0.59	2.98E−03	−0.074	0.015	1.05E−06	0.982	0.008	2.84E−02
rs11823949	11	47,723,512	AGBL2	A	0.35	1.98E−05	−0.097	0.017	4.40E−09	0.974	0.009	3.45E−03
rs35589443	2	127,847,736	BIN1	A	0.03	1.43E−03	−0.265	0.052	3.65E−07	1.047	0.027	9.53E−02
rs12419692	11	47,624,714	C1QTNF4	A	0.35	2.81E−05	−0.087	0.015	7.21E−09	0.977	0.008	5.11E−03
rs1317149	11	47,486,885	CELF1	T	0.36	2.17E−05	−0.089	0.015	3.45E−09	0.980	0.008	1.43E−02
rs11576522	1	207,789,269	CR1	A	0.63	2.85E−04	0.079	0.015	6.56E−08	1.017	0.009	5.87E−02
rs11039138	11	47,249,223	DDB2	A	0.37	9.00E−03	0.070	0.015	2.45E−06	1.015	0.008	7.91E−02
rs76184771	19	18,567,189	ELL	A	0.13	2.03E−02	−0.091	0.021	1.29E−05	1.030	0.011	9.58E−03
rs75010292	2	55,172,536	EML6	A	0.11	2.63E−02	−0.098	0.023	1.72E−05	0.967	0.013	7.70E−03
rs17125924	14	53,391,680	FERMT2	A	0.08	2.58E−03	−0.122	0.025	6.62E−07	1.026	0.014	5.79E−02
rs4742958	9	108,411,944	FKTN	T	0.14	4.44E−02	−0.128	0.030	1.56E−05	0.967	0.016	3.21E−02
rs7130758	11	47,749,960	FNBP4	T	0.36	3.27E−05	−0.088	0.015	1.01E−08	0.978	0.009	8.73E−03
rs11021857	11	11,472,193	GALNT18	A	0.07	4.92E−02	0.122	0.030	3.68E−05	1.049	0.017	4.60E−03
rs9270208	6	32,556,402	HLA-DRB1	T	0.68	4.29E−02	−0.077	0.018	1.49E−05	1.016	0.009	8.02E−02
rs2385088	19	18,545,540	ISYNA1	A	0.34	3.80E−02	−0.066	0.015	1.26E−05	1.016	0.008	6.29E−02
rs3859570	19	18,510,925	LRRC25	T	0.41	1.26E−02	−0.069	0.016	7.84E−06	1.023	0.009	9.53E−03
rs67871383	11	47,335,838	MADD	T	0.32	3.53E−04	0.082	0.015	8.28E−08	1.017	0.009	4.22E−02
rs2047007	1	40,427,975	MFSD2A	T	0.23	4.07E−02	0.071	0.017	2.83E−05	1.027	0.009	3.42E−03
rs1125357^b^	11	59,885,493	MS4A2	A	0.42	8.87E−09	0.107	0.015	3.09E−13	0.984	0.009	7.20E−02
rs1026254^b^	11	60,030,457	MS4A4A	T	0.60	2.84E−10	−0.116	0.015	2.42E−15	1.014	0.008	8.87E−02
rs1582763^b^	11	60,021,948	MS4A4E	A	0.36	1.40E−10	−0.123	0.015	1.19E−16	1.016	0.009	9.38E−02
rs624663^b^	11	59,945,065	MS4A6A	T	0.56	1.33E−08	0.104	0.014	6.34E−13	0.984	0.008	4.91E−02
rs10838738	11	47,663,049	MTCH2	A	0.35	2.56E−05	0.087	0.015	5.99E−09	1.025	0.008	2.82E−03
rs61781278	1	40,376,741	MYCL	A	0.19	4.30E−02	0.076	0.018	1.76E−05	1.022	0.010	2.47E−02
rs11605348	11	47,606,483	NDUFS3	A	0.35	3.47E−06	−0.097	0.016	4.25E−10	0.978	0.009	8.36E−03
rs7105282	11	47,805,614	NUP160	A	0.36	4.54E−05	−0.091	0.016	1.63E−08	0.974	0.009	3.15E−03
rs541458^b^	11	85,788,351	PICALM	T	0.68	1.01E−08	0.112	0.015	3.69E−13	0.984	0.010	8.07E−02
rs60075622	11	47,449,101	PSMC3	T	0.33	7.86E−06	0.092	0.015	1.30E−09	1.023	0.008	6.24E−03
rs755951	8	27,226,790	PTK2B	A	0.39	1.72E−04	−0.082	0.015	3.79E−08	1.015	0.008	6.82E−02
rs7106956	11	47,458,765	RAPSN	T	0.33	8.34E−06	0.092	0.015	1.28E−09	1.023	0.008	6.10E−03
rs755554	11	47,432,034	SLC39A13	C	0.67	4.90E−06	0.095	0.015	6.29E−10	1.024	0.009	4.71E−03
rs3781832	11	121,436,270	SORL1	T	0.10	2.89E−02	−0.098	0.022	1.09E−05	0.972	0.013	2.53E−02
rs67472071	11	47,391,745	SPI1	A	0.33	1.05E−06	−0.098	0.015	1.14E−10	0.980	0.008	1.96E−02
rs7258465	19	18,533,642	SSBP4	T	0.34	3.58E−02	−0.066	0.015	1.17E−05	1.017	0.008	4.36E−02
rs7011101	8	71,501,474	TRAM1	A	0.10	1.91E−02	−0.105	0.023	5.67E−06	1.022	0.013	9.91E−02
rs4929858	11	49,070,655	TRIM64C	A	0.63	2.80E−02	0.065	0.015	1.85E−05	1.036	0.008	1.85E−05
rs61781270	1	40,349,428	TRIT1	A	0.20	2.32E−02	0.077	0.017	7.11E−06	1.017	0.009	7.17E−02
rs66555393	8	71,628,753	XKR9	A	0.10	3.02E−02	0.106	0.024	9.54E−06	0.977	0.014	9.31E−02
rs17376517	3	44,765,221	ZNF502	T	0.05	4.63E−02	−0.141	0.033	1.65E−05	0.969	0.018	7.22E−02

*SNP* single-nucleotide polymorphism, *MAF* minor allele frequency, *LOAD* late-onset Alzheimer’s disease, *MDD* major depressive disorder, *FDR* false discovery rate.

^a^Build 37, assembly hg19.

^b^SNP that were highly significant (FDR *Q* ≤ 5 × 10^−8^) in the Q(LOAD|MDD) analysis excluding the APOE and were used for the eQTL analysis (Fig 4b).

The conditional association analysis in the reverse direction, *Q*(MDD|LOAD), exclusive of *APOE* region did not identify any SNP with FDR *Q* ≤ 0.05 (Fig. 2d). The strongest associated SNPs did not overlap with previously known GWAS LOAD loci (Table 2). Mapping genes based on proximity to the SNPs with the strongest conditional association for *Q*(MDD|LOAD), identified *TRMT61A*, *FOLH1*, *CKB* and *PTPRJ* and *PTPN1*.

**Table 2:** Genes reaching statistical significance (FDR ≤ 0.05) for association with MDD conditional on association with LOAD.

SNP	Chr	Position^a^	Nearest gene	Minor allele	MAF	Conditional FDR *Q*(MDD|LOAD)	LOAD Beta	LOAD Beta SE	LOAD *P*-value	MDD OR	MDD OR SE	MDD *P-*value
rs35591392	14	75,157,713	AREL1	T	0.53	2.82E–02	−0.047	0.014	8.83E−04	1.031	0.008	9.03E−05
rs78563874	11	85,609,073	CCDC83	T	0.24	2.76E–02	0.064	0.017	1.46E−04	0.964	0.009	9.09E−05
rs57105978	5	26,843,262	CDH9	T	0.03	2.02E–02	0.109	0.036	2.61E−03	0.930	0.019	1.20E−04
rs2071408	14	103,987,078	CKB	A	0.37	1.44E–02	0.049	0.015	1.12E−03	0.961	0.009	3.09E−06
rs7508819	20	49,205,320	FAM65C	T	0.58	1.94E–02	0.047	0.015	1.34E−03	1.031	0.008	1.79E−04
rs202675	11	49,228,613	FOLH1	C	0.28	1.79E–02	−0.054	0.016	7.43E−04	0.968	0.009	1.50E−04
rs74018295	15	57,870,174	GCOM1	T	0.09	1.90E–02	−0.077	0.026	3.13E−03	0.946	0.015	1.74E−04
rs111666866	6	32,491,925	HLA-DRB5	T	0.73	4.85E–02	0.060	0.018	8.22E−04	0.966	0.010	6.14E−04
rs4900582	14	104,058,014	KLC1	A	0.64	2.48E–02	0.052	0.016	8.84E−04	0.969	0.009	2.68E−04
rs8106047	19	18,505,741	LRRC25	A	0.16	4.31E–02	0.068	0.023	2.82E−03	0.941	0.014	1.30E−05
rs2894221	6	31,461,771	MICB	A	0.36	3.79E–02	−0.047	0.015	1.75E−03	0.968	0.008	7.42E−05
rs6664244	1	40,374,386	MYCL	T	0.36	2.47E–02	−0.057	0.015	1.32E−04	0.971	0.008	2.67E−04
rs7103992	11	48,510,777	OR4A47	A	0.65	2.84E–02	−0.045	0.015	2.26E−03	0.969	0.008	8.98E−05
rs10838830	11	48,233,932	OR4B1	A	0.35	2.42E–02	−0.044	0.015	3.06E−03	0.971	0.008	2.54E−04
rs4312096	11	48,411,653	OR4C5	C	0.65	1.93E–02	−0.045	0.015	2.66E−03	0.969	0.008	1.79E−04
rs3009872	1	66,411,400	PDE4B	T	0.43	3.87E–02	0.044	0.014	2.05E−03	1.028	0.008	4.68E−04
rs1265099	6	31,105,413	PSORS1C1	A	0.42	3.75E–02	0.048	0.015	1.07E−03	1.036	0.008	8.88E−06
rs6063534	20	49,189,168	PTPN1	T	0.51	1.80E–02	0.043	0.014	2.33E−03	1.031	0.008	1.36E−04
rs12790666	11	47,983,477	PTPRJ	A	0.52	1.79E–02	−0.045	0.014	1.71E−03	0.970	0.008	1.42E−04
rs12668707	7	4,414,425	SDK1	C	0.21	4.36E–02	−0.051	0.017	2.98E−03	1.034	0.010	5.38E−04
rs111653412	6	3,370,053	SLC22A23	T	0.02	2.38E–02	0.191	0.065	3.15E−03	0.863	0.040	2.47E−04
rs10157030	1	246,184,138	SMYD3	T	0.28	2.23E–02	0.056	0.016	5.73E−04	1.034	0.009	2.24E−04
rs6122859	20	48,610,609	SNAI1	A	0.63	3.99E–02	−0.047	0.015	2.12E−03	0.971	0.008	4.86E−04
rs7785189	7	12,257,527	TMEM106B	T	0.41	3.31E–02	−0.045	0.014	1.53E−03	1.032	0.008	7.77E−05
rs4268511	11	49,046,510	TRIM49B	A	0.64	1.81E–02	0.056	0.015	2.08E–04	1.032	0.008	1.32E−04
rs7949585	11	49,077,746	TRIM64C	T	0.60	2.00E–02	−0.051	0.015	6.11E–04	0.970	0.008	1.90E−04
rs911552	14	103,999,508	TRMT61A	A	0.37	8.94E–03	−0.051	0.015	5.98E–04	1.042	0.009	1.73E–06

*SNP* single-nucleotide polymorphism, *MAF* minor allele frequency, *LOAD* late-onset Alzheimer’s disease, *MDD* major depressive disorder, *FDR* false discovery rate.

^a^Build 37, assembly hg19.

The analyses were repeated for the data that included the *APOE* region. The conditional association analysis including the *APOE* region in which LOAD is conditional on association with MDD, *Q*(LOAD|MDD) showed an extremely strong signal, with FDR *Q* values ≤ 10^−120^ for the *APOE* region on chromosome 19 that was prominent on the Manhattan plot (Fig. 2a). The highly significant (FDR *Q* ≤ 5 × 10^–8^) cluster of SNPs on chromosome 11 also remained evident on the plot that included the *APOE* region. In the reverse condition *Q*(MDD|LOAD) analysis no SNPs were identified (FDR *Q* ≤ 1 × 10^−5^), however, with a relaxed FDR *Q* ≤ 0.05 (−log_10_(*Q*) = 1.3) numerous associated SNPs were found (Fig. 2b). Overall, the analysis of *Q*(LOAD|MDD) ≤ 0.05 including *APOE* region identified 458 SNPs representing 40 genes (Supplementary Table S1), where the corresponding analysis of the *Q*(MDD|LOAD) direction found 545 SNPs representing 27 genes (Supplementary Table S2).

Manhattan plots were also constructed for the conjunction conditional FDR (ccFDR) for the analysis that included the *APOE* region (Fig. 2e) and for an analysis that excluded the *APOE* region (Fig. 2f). The strongest association signals for the ccFDR showed marginal statistical significance for SNPs rs4929858 (FDR *Q* = 0.06) and rs3103780 (FDR *Q* = 0.07) that are proximal to the genes *TRIM49B* and *MYCL*, respectively.

### Mapping of genes to pathways and function

Eight out of the nine genes identified through the *Q*(LOAD|MDD) conditional analysis (FDR *Q* ≤ 0.05) were also found in LOAD-GWAS. These genes are involved in two major biological pathways and functional classes, immune response and regulation of endocytosis, that were previously implicated in LOAD by pathway analyses of GWAS data sets^55^. Specifically, *CR1*, *MS4A6A*, *SPI1*, and *CELF1* are relevant in immune response, and *BIN1*, *PICALM*, *SORL1*, and *PTK2B* in regulation of endocytosis.

Next, gene set enrichment was performed for the conditional FDRs in both directions, *Q*(LOAD|MDD) and *Q*(MDD|LOAD) using all SNPs. In this analysis, the SNP to gene mapping was limited to 500 kb upstream and downstream of the gene, and the candidate gene sets included canonical pathways, GO biological processes, and GO molecular function. The pathway gene set analysis using the LOAD conditional on MDD results showed the strongest statistical confidence (enrichment FDR < 0.05) for several biological pathways, including calcium channel activity, oxidoreductase activity acting on NLOADH or NLOADPH, receptor-mediated endocytosis, and phospholipid binding (Supplementary Table S3), following by hematopoietic cell lineage pathway with enrichment FDR = 0.04. The pathway mapping results for the receptor-mediated endocytosis were based on strong associations with 12 genes, from *PICALM* and *SORL1* that demonstrated the strongest effects, to *DMN1*, *IGF2R*, and *SFTPD* with weaker effects.

The significant pathways (FDR *Q* ≤ 0.05) identified using the results of the MDD conditional with LOAD represent a broad set of regulatory pathways, including leukocyte transendothelial migration, adherens junction, and purine metabolism (Supplementary Table S4). Notably, neurological system processes were identified as FDR significant (*P* ≤ 0.05).

Two genes showed association trends in the ccFDR analysis. One of which, *TRIM49B*, is a member of the TRIM family proteins which has a role in the innate immunity^56^, providing further evidence for the involvement of the immune system in LOAD.

### Gene expression analysis in brain

**Fig. 3:**
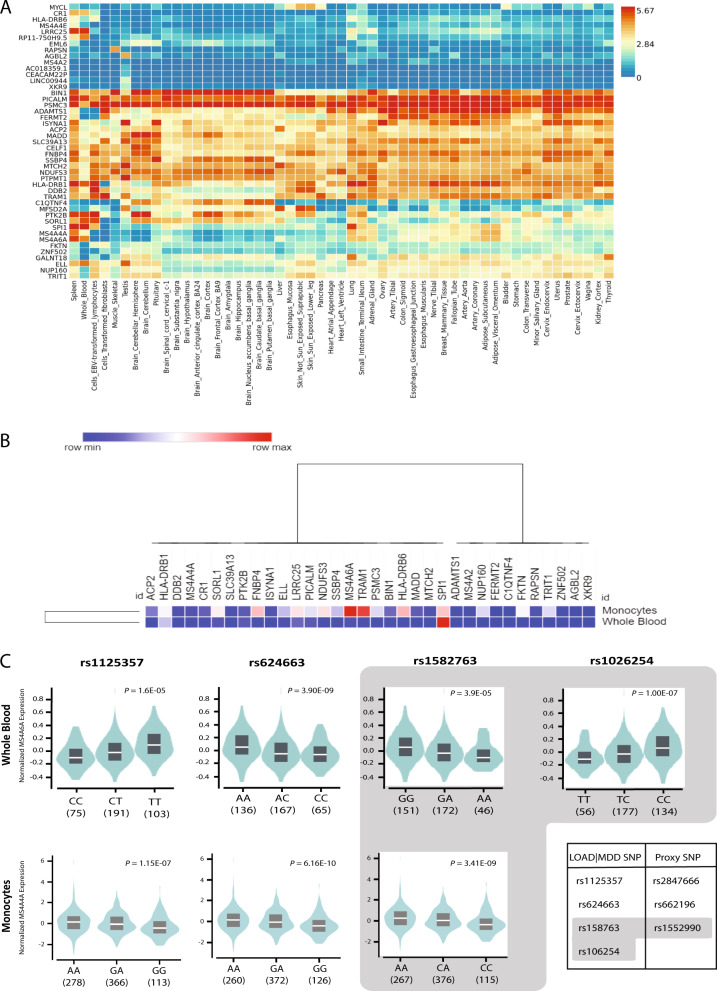
Gene expression and eQTL analysis for genes associated with LOAD conditional on association with MDD. **a** Heatmap plot of tissue-specific gene expression data in 53 GTEx tissue types for the 53 genes identified by proximity mapping for SNP association with LOAD, conditional with SNP association with MDD. There are 13 brain-specific tissues highlighted in the heatmap, which was ordered by both gene and tissue clustering. **b** Heatmap plot of expression levels of 53 genes in whole blood from the GTEx portal and in monocyte data from the Cardiogenics study. **c** Violin plots for eQTL analysis results in whole blood (upper panel) and in monocytes (lower panel) for SNPs identified as significant (FDR *Q* ≤ 5 × 10^−8^) for the association with LOAD conditional with association for MDD (Supplementary Table S5).

The tissue-specific gene expression for the 62 genes mapped by proximity to the SNPs identified in conditional association for *Q*(LOAD|MDD) was evaluated in 53 GTEx tissues (Fig. 3a). Fifty-three out of the total 62 genes were mapped to unique Entrez GeneID numbers, and are displayed in the heatmap. The heatmap was ordered by both gene and tissue clustering and denoted 13 brain-specific tissues. The *BIN1*, *PICALM*, and *PSMC2* genes showed high (5.7) levels of expression in the 13 brain tissues relative to all other genes. *MS4A4A*, *MS4A6A*, and *SPI1* showed a consistent, low expression level (~1.4) in the 13 brain tissues. *MTCH2*, *SSBP4*, and *PTPMT1* showed a consistent, moderate expression level (4.2) in all 13 brain tissues (Fig. 3a).

### eQTL analysis in myeloid lineage

We evaluated the set of 53 genes for expression levels in whole blood from the GTEx portal and for expression levels in monocyte data from the Cardiogenics study^53^. Only 34 genes had expression data in both dataset resources. We found six genes, including *MS4A6A* and *SPI1*, with high levels of expression relative to other genes in whole blood and in monocytes (Fig. 3b). The level of *SPI1* expression was higher than other genes in the set for the two tissues. The level of expression of *MS4A6A* was uniquely higher in monocytes than in whole blood (Fig. 3b).

Next, we performed an eQTL analysis for the five highly significant SNPs (FDR *Q* ≤ 5 × 10^−8^) on chromosome 11 (Table 2—marked in*; Supplementary Table S5), and found significant associations between the four SNPs clustered at the *MS4A* locus and mRNA levels of *MS4A6A* gene in whole blood (Fig. 3c; Supplementary Table S5). These four SNPs were not available in the monocytes expression data set; therefore, we conducted the eQTL analysis using proxy SNPs (*D*’ = 1, *R*^2^ > 0.9, Supplementary Table S5), and identified associations for all proxies with *MS4A4A* expression in monocytes (*P* < 5.4 × 10^−7^, Fig. 3c; Supplementary Table S5). SNP rs541458, proximal to *PICALM,* showed no significant eQTL signals in whole blood or in monocytes. SNP rs67472071 in the vicinity of *SPI1* was the next significantly associated with LOAD conditional on MDD (FDR *Q* = 1 × 10^−6^). We extended the eQTL analyses and found associations between this SNP and the expression of the regional *MYBPC3* and *C1QTNF4* genes in whole blood, and between its proxy and *MYBPC3* expression in monocytes (Supplementary Table S5).

## Discussion

This study represents the first step toward deciphering the genetic heterogeneity of NPS in LOAD, and in particular the comorbidity of depression and LOAD. The conditional FDR (cFDR) approach we undertook in this study was to identify genetic variants and genes that are associated with LOAD conditional on an association with MDD and vice versa. The cFDR approach showed clear precedent in a study that investigated overlap in variants associated with LOAD and plasma levels of c-reactive protein (CRP), low-density lipoprotein, high-density lipoprotein, and triglyceride. This previous study found polygenic overlap between LOAD and systemic inflammation measured by CRP and plasma lipids^28^. Moreover, by conditioning the LOAD association on inflammation and lipids phenotypes, they identified novel loci that were not reported in large LOAD case–control studies, and provided new insights into the involvement of pathways related to systemic inflammation, plasma lipids, and LOAD^28^. Similarly, another recent study examined the polygenic overlap between cognitive impairment and plasma CRP and lipids. They found an enrichment for SNPs associated with cognitive impairment conditional on plasma CRP and lipids, and significant associations for the *APOE* extended locus^36^. Shared genetic etiology for LOAD using the cFDR method was also investigated for Type 2 diabetes (T2D)^37^. In this study, multiple known and novel associated SNPs were identified by conditioning LOAD association on T2D and by the reverse direction, and mitochondrial dysfunction was highlighted as a common pathway^37^. The approach has been also utilized more broadly to examine genetic pleiotropy between multiple diverse diseases and phenotypes including schizophrenia and cognitive traits^29^, bipolar disorder^27^, multiple sclerosis^26^, cardiovascular disease risk factors^25^, and educational attainment^30^. Additional diseases and phenotypes to which the approach has been applied include Parkinson’s disease and autoimmune diseases^31^, blood lipids, immune-related diseases^32^ and more^33–35^. Collectively, using multiple disorders with overlapping phenotypes in genetic association studies allows the identification of shared genetic variants, genes, and pathways, which in turn elucidate common pathobiology and molecular mechanisms across disorders. Furthermore, conditioning genetic associations on multiple phenotypes is crucial to the discovery of novel loci that otherwise would not be identified via conventional case–control design.

In this study, we demonstrated shared genetic etiologies between LOAD and MDD. We observed a moderate enrichment for SNPs associated with LOAD across increasingly stringent levels of significance with MDD GWAS association, as well as in the reverse direction. Mendelian randomization analysis supported a potential causal relationship between the significant LOAD SNPs with MDD; this relationship was not a consequence of horizontal pleiotropy. In addition, we identified numerous associated SNPs and the biological interpretation of the corresponding genes pinpointed the immune response and regulation of endocytosis as common pathways. In contrast, Gibson et al. previously assessed overlapping polygenic architecture for LOAD and MDD and found no evidence for pleiotropy between these disorders^38^. These divergent outcomes may be explained by the different methodology used to investigate shared genetic between LOAD and MDD; while we applied a cFDR framework to detect genetic pleiotropy, the previous publication used LD score regression and polygenic risk score analysis. Alternatively, the different MDD cohorts from different countries may have been recruited using distinctive case diagnostic criteria, that may be anticipated given the arbitrary threshold criteria applied to clinical diagnosis of depression.

The LOAD genetic association analysis conditional on MDD *Q*(LOAD|MDD) showed an enrichment of SNPs on chromosome 11 with strong level of significance (Table 1). These SNPs are mapped predominantly in two major clusters on chromosome 11 that include genes previously implicated in LOAD–GWAS^16,20^. The first cluster features the *CELF1* and *SPI1* genes; while *CELF1* was the most proximate gene to the LOAD-SNP at this locus, *SPI1* was recently suggested as a stronger candidate causal gene for LOAD based on functional lines of evidence^57^. *SPI1* encodes PU.1, a transcription factor that is critical for myeloid cell development and function. A recent study reported an association between a SNP in the *SPI1* gene, rs1057233, and LOAD age of onset^57^. In addition, they performed an eQTL analysis and found that this SNP is associated with *SPI1* expression in monocytes and macrophages, suggesting that the encoded PU.1 may be a master regulator for the expression of multiple LOAD genes in myeloid cells^57^. Last, overexpression and downregulation of PU.1 in mouse microglial cells affected phagocytic activity and the expression of mouse orthologs of several LOAD risk genes. Based on these observations, we analyzed the linkage disequilibrium region of the *SPI1* locus. The SNP rs67472071 proximate to *SPI1* showed a conditional association for LOAD [*Q*(LOAD|MDD, FDR = 1.05 × 10^−6^)]. rs67472071 is located 15,297 base pairs from rs1057233 and is a proxy (*R*^2^ = 0.95 and *D*’ = 1) for this reported functional LOAD SNP rs1057233^57^. The second cluster of LOAD conditional MDD-associated SNPs encompasses the membrane-spanning 4-domains subfamily A (*MS4A*) genes region that encodes proteins with strong expression in the hematopoietic system. A new study integrated LOAD–GWAS with myeloid epigenomic and transcriptomic data sets to define candidate LOAD-risk enhancers in myeloid cells and their linked target causal genes^58^. By fine mapping of a candidate myeloid enhancer linked to the *MS4A* locus, they identified candidate functional SNP rs636317-T in the *MS4A* locus that affected the expression of *MS4A6A,* and validated it experimentally in human induced pluripotent stem cell (hiPSC)-derived microglia^58^. Consistent with this finding, our eQTL analysis showed that the cluster of SNPs at the *MS4A* locus are associated with the expression of *MS4A6A* in whole blood. Notable, in monocytes we showed that these SNPs were eQTLs for the adjacent *MS4A4A*. To further support the functional relevance of our finding, we examined the high LD region of the *MS4A* genes cluster, and found that two of the conditional LOAD-associated SNPs in this region, rs1582763 (proximal to *MS4A4E*) and rs1026254 (*MS4A4A*) located 2,798 and 11,307 base pairs away from the functional candidate SNP-rs636317, respectively, are proxies for this reported functional candidate SNP (*R*^2^ = 0.85 and *D*’ = 1, *R*^2^ = 1 and *D*’ = 1, respectively). Thus, the integrated data implies biological significance for the LOAD and MDD pleiotropic variants on chromosome 11 related to expression regulation in myeloid cells such as microglia.

Genetic studies implicated several genes on chromosome 11, including the *MS4A* cluster, *SPI1,* and *CELF1*, in both LOAD and MDD (GWAS summary statistics in Table 1). As discussed above, we identified LOAD and MDD pleiotropic variants proximal to these genes. The broader pleiotropic nature of these genes has not been studied extensively. A comprehensive literature search showed that these genes are also involved in non-neurological diseases (Supplementary Table S6). Interestingly, all of these neurological and non-neurological diseases are related, to varying extents, to the immune system. We interpret that the relationships of these genes to the various diseases may be exerted via their roles in immune response.

Our results warrant further in-depth investigations in several areas: (1) Confidence in the genetic association results is increased by the conditional association of two independent phenotypes, nevertheless future studies using replication cohorts will provide additional support to the results. (2) The conditional FDR framework identified associations of variants with the phenotypes. To advance our understanding of the causality, in this study we identified overlaps with eQTLs and suggested biological roles of the associated variants in regulation of gene expression. However, causality for the specific biological pathways would require validation studied using gene editing experiments in model systems. (3) Mapping of the associated SNPs to genes was inferred by proximity; other more distal relationships of the variants with genetic enhancers, for example, are important and ought to be evaluated experimentally using methods, such as Hi-C, to determine topographical associated domains (TAD). (4) The conditional FDR framework utilizes GWAS summary statistics from well-replicated consortium studies. Imputation of SNPs performed in these studies is based on 1000 Genomes reference panels, as is the pruning of SNPs by LD in the conditional FDR approach. Several studies have pointed to the limitations of using LD measures based on the 1000 genomes reference panel for genetic analysis using summary statistics, including conditional analysis, gene-based testing, fine mapping, and polygenic risk prediction^59–61^. As larger reference panels (e.g. Haplotype Reference Consortium^62^) and updated GWAS summary statistics based on these panels become available, the conditional analysis should also be updated.

In this study, we focused on LOAD and MDD to exemplify the concept of pleiotropy between two disorders that shared NPS. The results of this study provide a proof of concept for future work extending the evaluation of shared genetic etiology between LOAD and additional neuropsychiatric conditions that present comorbid NPS similar to those that manifest in LOAD. Moreover, further investigations are warranted to determine whether our observations are specific to LOAD and MDD, or can be generalized to other complex neuropsychiatric disorders with shared NPS.

In conclusion, the outcomes of this study have several important implications to LOAD genetic architecture, including the demonstration of genetic pleiotropy effects between LOAD and MDD, identification of new LOAD loci, and validation of LOAD pathways. Furthermore, our data propose a genetic interpretation of the heterogeneity of depression in LOAD. These findings are important for the development of actionable targets for novel therapies to treat depression preceding dementia, in an effort to delay or ultimately prevent the onset of LOAD.

## Supplementary information

Supplement Figure legends and Table titles

Figure S1. Manhattan plots of the GWAS results for LOAD and MDD.

Figure S2. Q-Q plots of the GWAS results for LOAD and MDD.

Supplemental Table S1. Summary statistics for analysis of LOAD conditional on MDD.

Supplemental Table S2. Summary statistics for analysis of MDD conditional on LOAD.

Supplemental Table S3. Pathway analysis for LOAD conditional on MDD.

Supplemental Table S4. Pathway analysis for MDD conditional on LOAD.

Supplemental Table S5. eQTL analysis of the top significant LOAD|MDD associated SNPs in whole blood and monocytes.

Supplemental Table S6. Diseases involved the top significant LOAD|MDD proximal genes.

## Data Availability

LOAD GWAS summary statistics: https://www.niagads.org/igap-rv-summary-stats-kunkle-p-value-data MDD GWAS summary statistics: https://www.med.unc.edu/pgc/results-and-downLOADs/mdd/

## Code availability

We have used the software cited in this paper with default parameters or minor changes, nonetheless, code for these analyses is available upon request.
