# Supplementary material for: Shared genetic etiology underlying Alzheimer’s disease and major depressive disorder
Source: Transl Psychiatry. 2020 Mar 9;10:88. doi: 10.1038/s41398-020-0769-y (PMC7062839; doi:10.1038/s41398-020-0769-y)
Supplement: Supplementary file 1 — Supplement Figure legends and Table titles [file 41398_2020_769_MOESM1_ESM.docx]

Figure. S1. **Manhattan plots of the GWAS results for LOAD and MDD.** (**A**) Manhattan plot for LOAD. (**B**) Manhattan plot for MDD. For both plots, the ordinate is –log_10_ of the *P* value for association for each SNP. Plots are made for results from the full genome. The red horizontal line is at the level of genome-wide significance, *P* ≤ 5×10^-8^, the blue horizontal line is at the level of suggestive significance, *P* ≤ 1×10^-5^.

Figure S2. **Q-Q plots of the GWAS results for LOAD and MDD.** (**A**) Q-Q plot for LOAD. (**B**) Q-Q plot for MDD. For both plots, the ordinate is –log_10_ of the observed *P* value and the abscissa is –log_10_ of the expected *P* value for association for each SNP.

Table S1. **Summary statistics for analysis of LOAD conditional on MDD.** Table provides SNP name, chromosome, coordinates for location, minor allele and minor allele frequency, nearest gene by proximity to the SNP, conditional FDR probability *Q*(LOAD|MDD) and summary statistics from the two individual GWAS, for LOAD: beta coefficient and standard error, *P* value; for MDD: Odds ratio and standard error and *P* value.

Table S2. **Summary statistics for analysis of MDD conditional on LOAD.** Table provides SNP name, chromosome, coordinates for location, minor allele and minor allele frequency, nearest gene by proximity to the SNP, conditional FDR probability *Q*(MDD|LOAD) and summary statistics from the two individual GWAS, for LOAD: beta coefficient and standard error, *P* value; for MDD: Odds ratio and standard error and *P* value.

Table S3. **Pathway analysis for LOAD conditional on MDD.** Pathway analysis was performed using *i*-Gsea4Gwas as described in Methods. Table provides name for the pathway/gene set and descriptor, gene set raw p value, FDR corrected p value for the gene set, number of significant genes, number of selected genes, all genes in the set and a link to the Gene Ontology description of the pathway/gene set.

Table S4. **Pathway analysis for MDD conditional on LOAD.** Pathway analysis was performed using *i*-Gsea4Gwas as described in Methods. Table provides name for the pathway/gene set and descriptor, gene set raw p value, FDR corrected p value for the gene set, number of significant genes, number of selected genes, all genes in the set and a link to the Gene Ontology description of the pathway/gene set.

Table S5. **eQTL analysis of the top significant LOAD|MDD associated SNPs in whole blood and monocytes**

Table S5. **Diseases involved the top significant LOAD|MDD proximal genes**
