# Supplementary figures and images for: Shared genetic etiology underlying Alzheimer’s disease and major depressive disorder
Source: Transl Psychiatry. 2020 Mar 9;10:88. doi: 10.1038/s41398-020-0769-y (PMC7062839; doi:10.1038/s41398-020-0769-y)

Supplemental Fig. S1. Manhattan plots of the GWAS results for LOAD and MDD

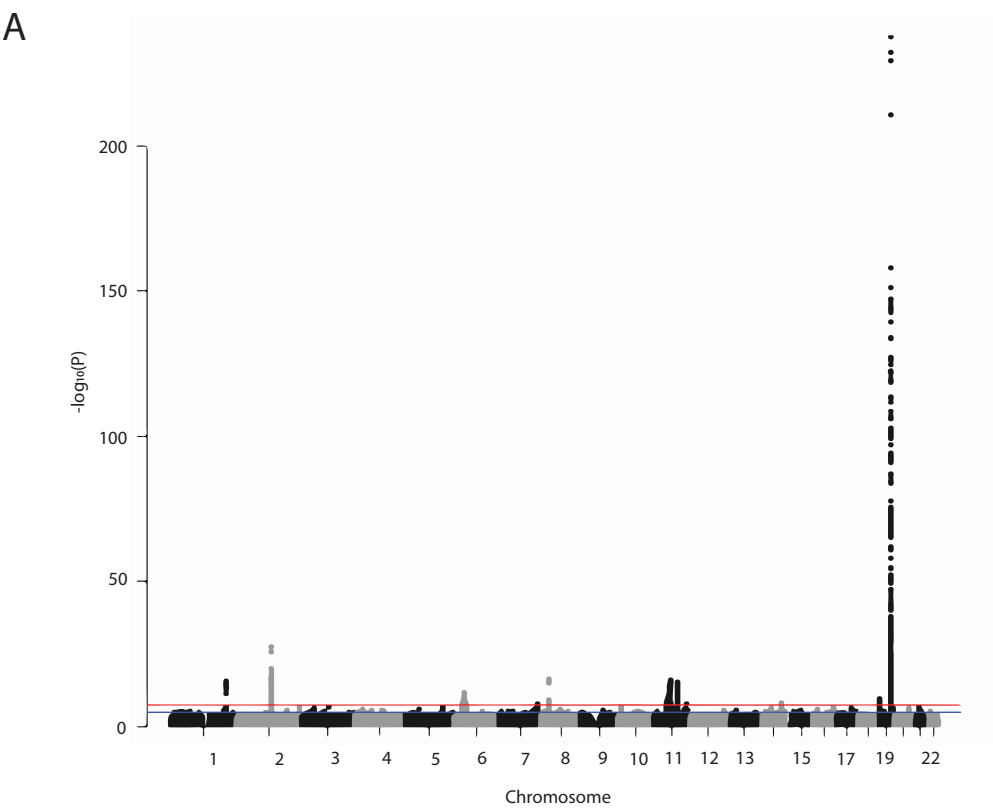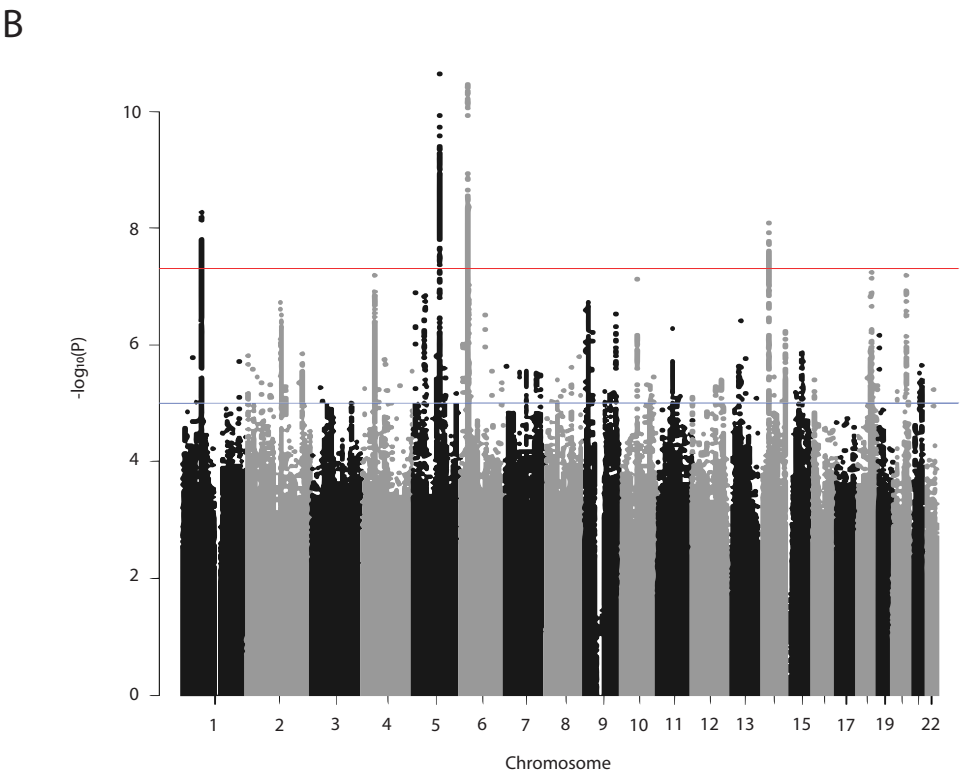

Supplement: Supplementary file 2 — Figure S1. Manhattan plots of the GWAS results for LOAD and MDD. [file 41398_2020_769_MOESM2_ESM.pdf]

Supplemental Fig. S2:. Q-Q plots of the GWAS results for LOAD and MDD.

A

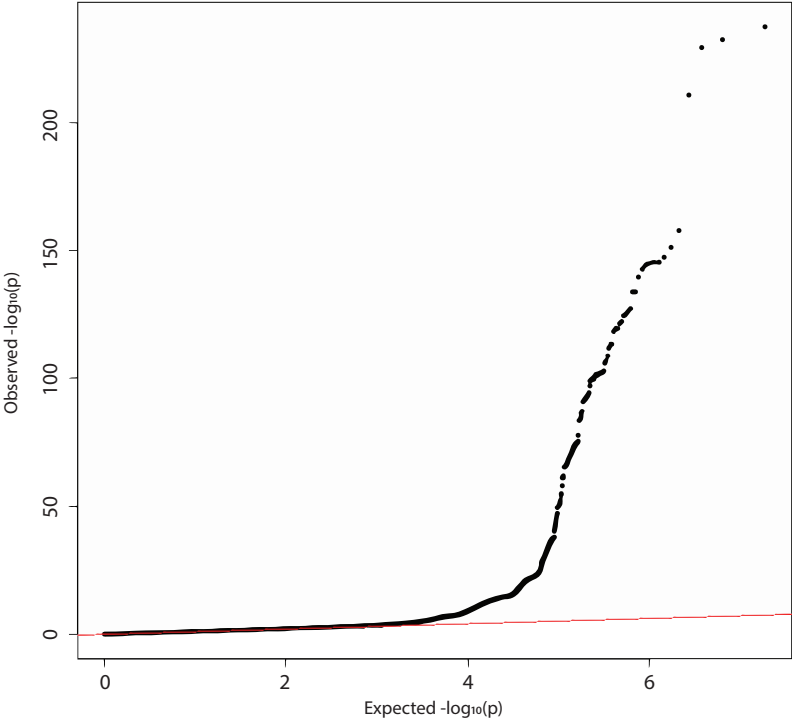

B

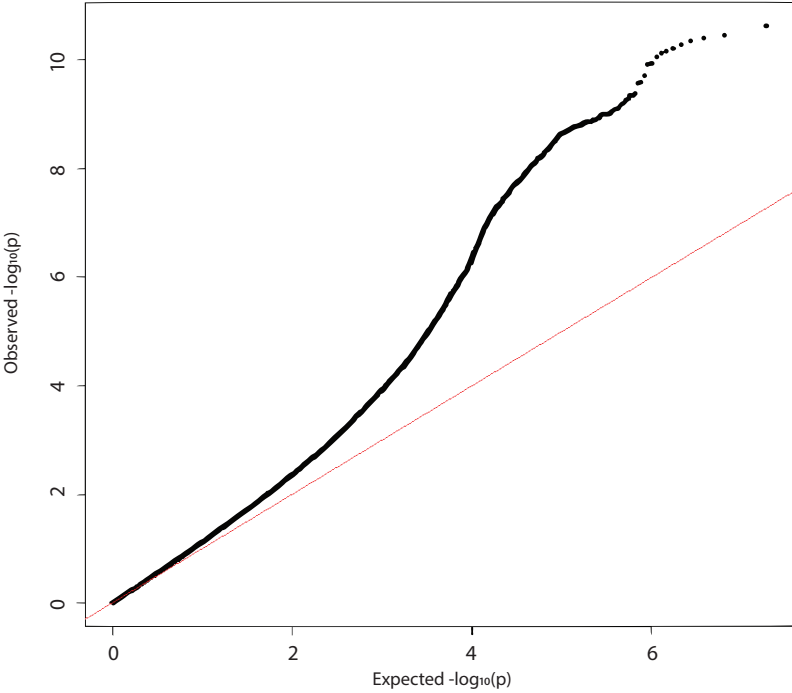

Supplement: Supplementary file 3 — Figure S2. Q-Q plots of the GWAS results for LOAD and MDD. [file 41398_2020_769_MOESM3_ESM.pdf]
