# Supplementary material for: Shared genetic etiology underlying Alzheimer’s disease and major depressive disorder
Source: Transl Psychiatry. 2020 Mar 9;10:88. doi: 10.1038/s41398-020-0769-y (PMC7062839; doi:10.1038/s41398-020-0769-y)
Supplement: Supplementary file 8 — Supplemental Table S5. eQTL analysis of the top significant LOAD|MDD associated SNPs in whole blood and monocytes. [file 41398_2020_769_MOESM8_ESM.docx]

**Supplemental Table S5. eQTL analysis of the top significant LOAD|MDD associated SNPs in whole blood and monocytes**

| **LOAD\|MDD SNP** | | | **Whole Blood^b^** | | **Monocytes^c^** | | | | | |
| --- | --- | --- | --- | --- | --- | --- | --- | --- | --- | --- |
| **SNP ID** | **Coordinates^a^** | **Closest gene** | **eQTL**  **gene** | **eQTL *P*value** | **Proxy ID** | **Distance**^d^ | **D’** | **r^2^** | **eQTL gene** | **eQTL *P*value^b^** |
| rs1582763 | chr11:60,021,948 | MS4A4E | MS4A6A | 0.000039 | rs1562990 | 1139 | 1 | 0.8108 | MS4A4A | 3.41E-09 |
| rs1026254 | chr11:60,030,457 | MS4A4A | MS4A6A | 1.00E-07 | rs1562990 | -7370 | 1 | 0.9892 | MS4A4A | 3.41E-09 |
| rs1125357 | chr11:59,885,493 | MS4A2 | MS4A6A | 0.000016 | rs2847666 | -25917 | 1 | 0.9576 | MS4A4A | 1.15E-07 |
| rs624663 | chr11:59,945,065 | MS4A6A | MS4A6A | 3.90E-09 | rs662196 | -2308 | 1 | 1 | MS4A4A | 6.16E-10 |
| rs67472071 | chr11:47,391,745 | SPI1 | C1QTNF4 | 2.60E-16 | rs10769258 | -706 | 1 | 1 | MYBPC3 | 5.85E-15 |
|  |  |  | MYBPC3 | 6.70E-12 |  |  |  |  |  |  |

^a^ Build 37, assembly hg19.

^b^ GTEx portal v7

^c^ Cardiogenics

^d^ Distance (bp) from LOAD|MDD associated SNP (Build 37, assembly hg19). (-), downstream of the associated SNP.
