# Supplementary material for: Shared genetic etiology underlying Alzheimer’s disease and major depressive disorder
Source: Transl Psychiatry. 2020 Mar 9;10:88. doi: 10.1038/s41398-020-0769-y (PMC7062839; doi:10.1038/s41398-020-0769-y)
Supplement: Supplementary file 9 — Supplemental Table S6. Diseases involved the top significant LOAD|MDD proximal genes. [file 41398_2020_769_MOESM9_ESM.docx]

| **Gene** | **Suggested General Function** | **Associated diseases** |
| --- | --- | --- |
| MS4A4E | May be involved in regulation of calcium signaling | No reported disorders |
| MS4A4A | May be involved in regulation of calcium signaling | No reported disorders |
| MS4A2 | Allergic response, protein kinase binding, secretion of lymphokines | IgE responsiveness, atopic ^1^ |
|  |  | allergic asthma^2^ |
|  |  | [asthma](http://www.malacards.org/card/asthma)^3^ |
|  |  | chronic rhinitis^4^ |
|  |  | allergic rhinitis^5^ |
|  |  | [dermatitis, atopic](http://www.malacards.org/card/dermatitis_atopic)^6^ |
| MS4A6A | Regulation of calcium signaling as a component of a multimeric receptor complex. | [hydrocephalus, normal-pressure](http://www.malacards.org/card/hydrocephalus_normal_pressure)^7,8^ |
| SPI1 | DNA-binding transcription factor, RNA binding, pre-mRNA splicing | [primary mediastinal b-cell lymphoma](http://www.malacards.org/card/primary_mediastinal_b_cell_lymphoma) |
|  |  | erythroleukemia, familial^9^ |
|  |  | poikiloderma with neutropenia^10^ |
|  |  | [T-cell leukemia](http://www.malacards.org/card/t_cell_leukemia)^11^ |
|  |  | [microphthalmia](http://www.malacards.org/card/microphthalmia)^12^ |
|  |  | acute promyelocytic leukemia^13^ |
|  |  | [leukemia, acute myeloid](http://www.malacards.org/card/leukemia_acute_myeloid)^14^ |
| CELF1 | RNA binding, pre-mRNA splicing, mRNA editing | myotonic dystrophy type 1^15^ |

**Supplemental Table S6. Diseases involved the top significant LOAD|MDD proximal genes**

**REFERENCES**

1 Sandford, A. J. *et al.* Localisation of atopy and beta subunit of high-affinity IgE receptor (Fc epsilon RI) on chromosome 11q. *Lancet* **341**, 332-334, doi:10.1016/0140-6736(93)90136-5 (1993).

2 March, M. E., Sleiman, P. M. & Hakonarson, H. Genetic polymorphisms and associated susceptibility to asthma. *Int J Gen Med* **6**, 253-265, doi:10.2147/IJGM.S28156 (2013).

3 Traherne, J. A. *et al.* LD mapping of maternally and non-maternally derived alleles and atopy in FcepsilonRI-beta. *Hum Mol Genet* **12**, 2577-2585, doi:10.1093/hmg/ddg290 (2003).

4 Palikhe, N. S., Kim, J. H. & Park, H. S. Update on recent advances in the management of aspirin exacerbated respiratory disease. *Yonsei Med J* **50**, 744-750, doi:10.3349/ymj.2009.50.6.744 (2009).

5 Amo, G. *et al.* A Nonsynonymous FCER1B SNP is Associated with Risk of Developing Allergic Rhinitis and with IgE Levels. *Sci Rep* **6**, 19724, doi:10.1038/srep19724 (2016).

6 Lee, Y. A. *et al.* A major susceptibility locus for atopic dermatitis maps to chromosome 3q21. *Nat Genet* **26**, 470-473, doi:10.1038/82625 (2000).

7 Proitsi, P. *et al.* Alzheimer's disease susceptibility variants in the MS4A6A gene are associated with altered levels of MS4A6A expression in blood. *Neurobiol Aging* **35**, 279-290, doi:10.1016/j.neurobiolaging.2013.08.002 (2014).

8 Huovinen, J. *et al.* Alzheimer's Disease-Related Polymorphisms in Shunt-Responsive Idiopathic Normal Pressure Hydrocephalus. *J Alzheimers Dis* **60**, 1077-1085, doi:10.3233/JAD-170583 (2017).

9 Schuetze, S., Paul, R., Gliniak, B. C. & Kabat, D. Role of the PU.1 transcription factor in controlling differentiation of Friend erythroleukemia cells. *Mol Cell Biol* **12**, 2967-2975, doi:10.1128/mcb.12.7.2967 (1992).

10 Colombo, E. A. *et al.* A zebrafish model of Poikiloderma with Neutropenia recapitulates the human syndrome hallmarks and traces back neutropenia to the myeloid progenitor. *Sci Rep* **5**, 15814, doi:10.1038/srep15814 (2015).

11 Tsukada, J. *et al.* Human T-cell leukemia virus type I Tax transactivates the promoter of human prointerleukin-1beta gene through association with two transcription factors, nuclear factor-interleukin-6 and Spi-1. *Blood* **90**, 3142-3153 (1997).

12 So, H. *et al.* Microphthalmia transcription factor and PU.1 synergistically induce the leukocyte receptor osteoclast-associated receptor gene expression. *J Biol Chem* **278**, 24209-24216, doi:10.1074/jbc.M302940200 (2003).

13 Delestre, L. *et al.* Senescence is a Spi1-induced anti-proliferative mechanism in primary hematopoietic cells. *Haematologica* **102**, 1850-1860, doi:10.3324/haematol.2016.157636 (2017).

14 Abo Elwafa, R., Gamaleldin, M. & Ghallab, O. The clinical and prognostic significance of FIS1, SPI1, PDCD7 and Ang2 expression levels in acute myeloid leukemia. *Cancer Genet* **233-234**, 84-95, doi:10.1016/j.cancergen.2018.12.001 (2019).

15 Timchenko, L. Correction of RNA-Binding Protein CUGBP1 and GSK3beta Signaling as Therapeutic Approach for Congenital and Adult Myotonic Dystrophy Type 1. *Int J Mol Sci* **21**, doi:10.3390/ijms21010094 (2019).
